# Mississippi Floods

**Published:** 1882-11

**Authors:** 


### MISSISSIPPI FLOODS.

It is said by those who seem to be most competent to judge, that no practicable system of levees ever yet devised could restrain the waters of the Mississippi from overflowing during certain seasons of the year.

One authority says that it would require levees three hundred feet high, not only along the great river, but along its tributaries also. This is probably an exaggeration; but it shows that in his opinion the whole system is impracticable.

We have a plan of our own which we have submitted to several well-known engineers of our acquaintance, which, whatever its merits, has elicited a good deal of attention on the part of these gentlemen. Our plan, whether good, bad or indifferent, is, so far as we are aware, entirely original; although, now that we have divulged it, we are prepared to hear that Thomas, Richard and Henry thought it all over forty years ago.

No competent physician would attempt to cure a plethoric patient by bleeding him; but he would check the supply of blood by lessening the quantity of material from which blood is made. No "River and Harbor bill" quackery can ever control the floods after they have been allowed to reach the bed of the mighty river. Our surgery contemplates cutting off the supplies before they reach the Mississippi, so that not enough water will reach its channel to produce an overflow; and this we would do by diverting a portion of the waters of its tributaries and causing them to flow into the Gulf of Mexico through direct channels instead of flowing into the Mississippi. By reference to a map of the Mississippi Valley, it will be observed that there are many streams of various sizes, whose general course is parallel to the great river, and into which most of them finally empty. Now if, instead of allowing these streams to run into the Mississippi they were to be cut off, and artificial channels were to be dug to connect each stream with the stream below it, so that the last stream to receive the waters thus accumulated should be the Atchafalaya River, which runs a straight and short course to the Gulf of Mexico, the result would probably be, to divert enough water from the Mississippi to prevent overflows, and, perhaps, to form another important water course for commerce.

If one such channel is not sufficient to accomplish the object, the topography of that country is favorable to the construction of any number of similar channels; and enough tributaries could thus be connected, and enough water diverted from natural channels to accomplish the object with far greater certainty and more economy than any system of levees. It is probable that the improved sanitary condition of that section of country, the reclamation of waste lands, and the increased commerce which the improvement in the water courses would attract, would of themselves compensate for the entire outlay.

All details of the plan as to locks, grades and other matters come within the province of the engineer; but the plan itself we shall claim as our own unless some one else can show a better title.

